# The validity of neoadjuvant chemotherapy with paclitaxel plus S-1 is not inferior to that of SOX regimen for locally advanced gastric cancer: an observational study

**DOI:** 10.1186/s12885-022-10230-1

**Published:** 2022-11-28

**Authors:** Chenghai Zhang, Binghong Wu, Hong Yang, Zhendan Yao, Nan Zhang, Fei Tan, Maoxing Liu, Kai Xu, Lei Chen, Jiadi Xing, Ming Cui, Xiangqian Su

**Affiliations:** Key Laboratory of Carcinogenesis and Translational Research (Ministry of Education), Department of Gastrointestinal Surgery IV, Peking University Cancer Hospital & Institute, 52 Fu-Cheng Road, Hai-Dian District, Beijing, 100142 China

**Keywords:** Locally advanced gastric cancer, Neoadjuvant chemotherapy, Pathological response, Oxaliplatin, Paclitaxel

## Abstract

**Background:**

Paclitaxel plus S-1(PTXS) has shown definite efficacy for advanced gastric cancer. However, the efficacy and safety of this regimen in neoadjuvant setting for locally advanced gastric cancer (LAGC) are unclear. This study aimed to compare the efficacy of neoadjuvant chemotherapy (NAC) PTXS and oxaliplatin plus S-1 (SOX) regime for patients with LAGC.

**Methods:**

A total of 103 patients with LAGC (cT3/4NanyM0/x) who were treated with three cycles of neoadjuvant SOX regimen (*n* = 77) or PTXS regimen (*n* = 26) between 2011 and 2017 were enrolled in this study. NAC-related clinical response, pathological response, postoperative complication, and overall survival were analyzed between the groups.

**Results:**

The baseline data did not differ significantly between both groups. After NAC, the disease control rate of the SOX group (94.8%) was comparable with that of the PTXS group (92.3%) (*p* = 0.641). Twenty-three cases (29.9%) in the SOX group and 10 cases (38.5%) in the PTX group got the descending stage with no statistical difference (*p* = 0.417). No significant differences were observed in the overall pathological response rate and the overall postoperative complication rate between the two groups (*p* > 0.05). There were also no differences between groups in terms of 5-year overall and disease-free survival (*p* > 0.05).

**Conclusions:**

The validity of NAC PTXS was not inferior to that of SOX regimen for locally advanced gastric cancer in terms of treatment response and overall survival. PTXS regimen could be expected to be ideal neoadjuvant chemotherapy for patients with LAGC and should be adopted for the test arm of a large randomized controlled trial.

**Supplementary Information:**

The online version contains supplementary material available at 10.1186/s12885-022-10230-1.

## Introduction

Compared with early gastric cancer, the current treatment of LAGC is still challenging worldwide. Although adjuvant chemotherapy can reduce the risk of recurrence and prolong survival in patients undergoing gastrectomy with D2 lymphadenectomy, the overall 5-year survival rate of patients remains low [1]. Therefore, since the MAGIC (Medical Research Council Adjuvant Gastric Infusional Chemotherapy) trial, numerous clinical studies on neoadjuvant chemotherapy for gastric cancer have been carried out [1–5]. The advantages of neoadjuvant chemotherapy, including tumor downstaging, improved R0 resection rate, validation of drug sensitivity, reduced recurrence, and improved survival, have been confirmed by more and more studies [1, 2, 4].

Neoadjuvant chemotherapy regimens for LAGC mainly refer to adjuvant chemotherapy regimens, including platinum-containing regimens and taxane-containing regimens. A series of large German multicenter clinical studies showed that the superiority of the neoadjuvant FLOT regimen (docetaxel plus oxaliplatin, fluorouracil, and leucovorin) over the ECF or ECX regimens (epirubicin plus cisplatin, and fluorouracil/capecitabine) in terms of overall survival and pathological response [6, 7]. Therefore, the FLOT regimen has become the standard regimen for perioperative chemotherapy for LAGC in European countries. However, the S-1-based doublet regimens (S-1 plus platinum, S-1 plus oxaliplatin, S-1 plus paclitaxel) are commonly used in perioperative chemotherapy regimens in Asian countries, and numerous studies have demonstrated the efficacy and safety of these doublet regimens [8–11]. The Chinese large-scale phase III clinical trial (RESOLVE) confirmed the significant efficacy of the neoadjuvant SOX (S-1 plus oxaliplatin) regimen, and thus established the SOX regimen as the first choice for neoadjuvant chemotherapy for LAGC in China [12]. In addition, a recent randomized clinical trial reported that there were no significant differences in complete or subtotal tumor regression grading, adverse effects, and postoperative morbidity between the neoadjuvant FLOT group and the SOX group [13].

The efficacy of the taxane-based triplet regimen in perioperative chemotherapy for LAGC was confirmed, but this regimen was considered more toxic [7, 13, 14]. Therefore, a taxane-based doublet regimen such as PTXS was also used as neoadjuvant chemotherapy for patients with LAGC. And several studies showed that the neoadjuvant PTXS regimen was effective and safe for patients with LAGC [11, 15]. Moreover, a high-quality review concluded that the PTXS chemotherapy was more effective and safer for advanced gastric cancer when compared with S-1 plus other drugs or S-1 alone [16]. Therefore, the neoadjuvant PTXS chemotherapy was also used for patients with LAGC in our center, with good effects and low side effects.

To date, there are no previous studies on neoadjuvant chemotherapy for patients with LAGC that compared the safety and efficacy of the SOX and PTXS regimens. So, we performed this retrospective study to explore the difference in clinical response, pathological response, postoperative complication, and overall survival between the two regimens.

## Patients and methods

### Patients

**Fig. 1:**
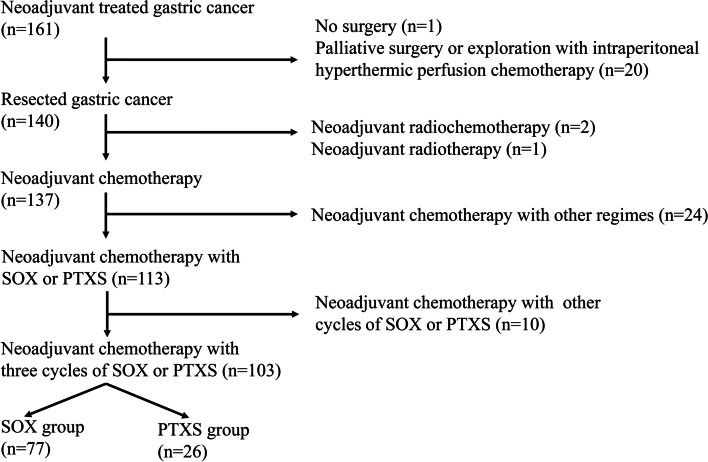
Eligible patients included in this study

A total of 161 patients with locally advanced gastric adenocarcinoma (cT3/4NanyM0/x) received neoadjuvant chemotherapy between 2011 and 2017 in our center (Department of Gastrointestinal Surgery IV, Peking University Cancer Hospital & Institute). Among them, 103 patients who were treated with three cycles of neoadjuvant SOX regimen (*n* = 77) or PTXS regimen (*n* = 26) were included in the present study (Fig. 1). There was no uniform standard for neoadjuvant chemotherapy for LAGC, which mainly depended on the doctor's preference for medication or the potential side effects of chemotherapy drugs.

Clinicopathological data were collected in a prospectively generated database. Written informed consent was obtained from each patient enrolled in the study. All operations in this study were in accordance with the ethical standards of the Ethics Committee of Peking University Cancer Hospital & Institute.

### Treatment

The neoadjuvant SOX chemotherapy was administered to patients with 3 cycles (3 weeks per cycle) of intravenous oxaliplatin (130 mg/m^2^ on day 1) plus oral S-1 (40-60 mg/m^2^ twice daily on days 1 to 14), if there were no intolerable side effects. Day 15 to day 21 was the rest week. Similarly, the neoadjuvant PTXS regimen was given to patients with 3 cycles (3 weeks per cycle) of intravenous paclitaxel (175 mg/m^2^ on day 1) plus oral S-1 (40-60 mg/m^2^ twice daily on days 1 to 14). The drug dose was adjusted according to patients with grade three and above adverse effects.

Lesions were evaluated according to the Response Evaluation Criteria in Solid Tumors (RECIST 1.1) criteria by enhanced CT, endoscopic ultrasonography (EUS), and MRI as needed after 3 cycles of neoadjuvant chemotherapy. Laparoscopic or open radical gastrectomy with standard D2 lymphadenectomy was performed 3–4 weeks after the last cycle of neoadjuvant chemotherapy. The extent of gastric resection and lymph node dissection was performed according to the gastric treatment guidelines [17].

All patients started to receive adjuvant chemotherapy about one month after surgery, and the regimen was usually continued with the preoperative regimen except for disease progression. The duration of perioperative chemotherapy was half a year. Dose reduction or chemotherapy discontinuation was carried out to help patients cope with serious side effects. Oral S-1 alone could be continued as monotherapy when patients could not tolerate the severe adverse events caused by the combined chemotherapy.

### Follow-up

Patients received examinations every 3 months for the first two years after surgery, every 6 months for the next 3 years, and every year thereafter. Examinations included physical examinations, laboratory tests, X-ray/CT of the chest, and CT/ultrasonography of the abdomen and pelvis. PET/CT was conducted when appropriate.

### The evaluation of the clinical response

The primary gastric lesion and perigastric lymph nodes examined by the contrast-enhanced CT were assessed by professional radiologists using Response Evaluation Criteria in Solid Tumors (RECIST; version 1.1) [18]. Complete response (CR) means that all target lesions have disappeared. Partial response (PR) is defined as at least a 30% decrease in the sum of diameters of all target lesions compared with the baseline sum diameters. Progressive disease (PD) refers to the appearance of one or more new lesions or at least a 20% increase in the sum of diameters of target lesions. Stable disease (SD) is defined as neither a sufficient decrease to qualify for PR nor a sufficient increase to qualify for PD. Disease control rate (DCR) represents the sum of CR, PR, and SD rates.

### The evaluation of the pathological assessment

The degree of pathological response was classified according to TRG criteria. TRG 0 (complete response): no viable cancer cells, including lymph nodes; TRG 1 (near-complete response): single cells or rare small groups of cancer cells; TRG 2 (partial response): residual cancer cells with evident tumor regression but more than single cells or rare groups of cancer cells; TRG 3 (poor or no response): extensive residual cancer with no evident tumor regression.

### Statistical analysis

Categorical variables were analyzed using the Chi-square test or Fisher’s exact test. Continuous variables were compared with *t*-tests or Mann–Whitney U tests. The overall survival (OS) was defined as the period from the time of operation to death or last follow-up. The disease-free survival (DFS) was defined as the period from the time of operation to recurrence. Survival curves were constructed by the Kaplan–Meier method, and differences were analyzed by the log-rank tests. Statistical analyses were calculated by SPSS version 23.0 (SPSS, Inc., Chicago, IL, USA). *P*-value < 0.05 was considered significant.

## Results

### Patient and clinical characteristics

During the study period, 161 patients with LAGC received neoadjuvant therapy. A total of 87 cases were excluded from the study for various reasons (Fig. 1). Finally, 103 eligible patients (77 in the SOX group and 26 in the PTXS group) received three cycles of neoadjuvant chemotherapy and underwent gastrectomy with D2 lymphadenectomy. No significant differences were observed in age, gender, body mass index, tumor location, cT stage, cN stage, and cTNM stage between the SOX and PTXS groups (Table 1, *p* > 0.05). All cases received D2 lymphadenectomy, 67.5% of patients in the SOX group and 65.4% of patients in the PTXS group underwent total gastrectomy. A total of 79.2% versus 80.8% of patients were diagnosed as stage III in the SOX and PTXS groups, respectively. Therefore, the baseline data of the two groups of patients were balanced.

**Table 1:** Patients baseline characteristics between both groups

**Parameter**	**SOX (n, %)**	**PTXS (n, %)**	***P*** **value**
**Sex**			0.114
**Male**	62 (80.5)	17 (65.4)	
**Female**	15 (19.5)	9 (34.6)	
**Age (years)**			0.835
**Median (range)**	59 (24–74)	61 (29–74)	
**Body mass index (kg/m**^**2**^**)**			0.866
**Median (range)**	22.4 (15.4–33.6)	22.8 (16.1–22.8)	
**Tumor location**			0.662
**Proximal part**	41 (53.2)	11 (42.3)	
**Middle part**	12 (15.6)	6 (23.1)	
**Distal part**	21 (27.3)	7 (26.9)	
**Diffuse**	2 (3.9)	2 (7.7)	
**cT stage (pre-NAC)**			0.737
**T3**	12 (15.6)	5 (19.2)	
**T4**	65 (84.4)	21 (80.8)	
**cN stage (pre-NAC)**			0.083
**N0**	4 (5.2)	2 (7.7)	
**N1**	33 (42.9)	10 (38.5)	
**N2**	34 (44.2)	7 (26.9)	
**N3**	6 (7.7)	7 (26.9)	
**cTNM stage (pre-NAC)**			0.865
**II**	16 (20.8)	5 (19.2)	
**III**	61 (79.2)	21 (80.8)	
**Scope of gastrectomy**			0.84
**Partial**	25 (32.5)	9 (34.6)	
**Total**	52 (67.5)	17 (65.4)	

*NAC* Neoadjuvant chemotherapy, *SOX* Oxaliplatin plus S-1, *PTXS* Paclitaxel plus S-1

### Radiological response

No significant difference was observed in the pretreatment cTNM stage between the PTXS group and the SOX group. Among 77 patients in the SOX group, 0, 33, 40, and 4 cases, respectively, obtained CR (0%), PR (42.9%), SD (51.9%) and PD (5.2%). Among 26 patients in the PTX group, 0,9,15, and 2 cases, respectively, received CR (0%), PR (34.6%), SD (57.7%) and PD (7.7%) (Table 2). From Table 2, the disease control rate (CR + PR + SD) of the SOX group (94.8%) was comparable with that of the PTXS group (92.3%) (*p* = 0.641).

**Table 2:** Radiological evaluation by computed tomography after neoadjuvant chemotherapy

**Parameter**	**SOX group**	**PTXS group**	***P*** **value**
**T stage (post-NAC)**			0.088
**T1**	1 (1.3)	0 (0.0)	
**T2**	2 (2.6)	0 (0.0)	
**T3**	33 (42.9)	18 (69.2)	
**T4**	41 (53.2)	8 (30.8)	
**N stage (post-NAC)**			0.370
**N0**	5 (6.5)	4 (15.4)	
**N1**	50 (64.9)	14 (53.8)	
**N2**	20 (26.0)	6 (23.1)	
**N3**	2 (2.6)	2 (7.7)	
**TNM stage (post-NAC)**			0.190
**II**	33 (42.9)	15 (57.7)	
**III**	44 (57.1)	11 (42.3)	
**Response rate**			0.188
**CR**	0 (0.0)	0 (0.0)	
**PR**	33 (42.9)	9 (34.6)	
**SD**	40 (51.9)	15 (57.7)	
**PD**	4 (5.2)	2 (7.7)	
**DCR (CR + PR + SD)**	73 (94.8)	24 (92.3)	0.641
**Descending stage rate**	23 (29.9)	10 (38.5)	0.417

*NAC* Neoadjuvant chemotherapy, *SOX* Oxaliplatin plus S-1, *PTXS* Paclitaxel plus S-1, *PR* Partial response, *SD* Stable disease, *PD* Progressive disease, *DCR* Disease control rate

### Descending stage rate

After neoadjuvant chemotherapy, a total of 33 patients (32%) got the descending stage. There were 23 cases (29.9%) in the SOX group and 10 cases (38.5%) in the PTX group with no statistical difference (*p* = 0.417) (Table 2).

### Pathological response

There was no significant difference in the R0 resection rate between the SOX group (93.5%) and the PTXS group (92.3%). According to Lauren’s classification, 16.9% of tumors in the SOX group and 26.9% of tumors in the PTXS group were diffuse types. The proportion of the N0 stage was relatively higher in the SOX group (48%) than in the PTXS group (34.6%), but there was no significant difference between both groups. Compared with the pre-neoadjuvant chemotherapy (pre-NAC) T and N stage, the pathological T and N stages decreased significantly in both groups (Tables 2 and 3). The downstaging rate of the T stage in the SOX group was significantly better than that in the PTXS group (*p* = 0.015). The median number of harvested lymph nodes was 26 in the SOX group and 32 in the PTXS group with no significant difference (Table 3, *p* = 0.120).

**Table 3:** Clinicopathological results of two groups

**Parameter**	**SOX (n, %)**	**PTXS (n, %)**	***P* value**
**Lauren's classification**			0.052
**Intestinal**	42 (54.5)	7 (26.9)	
**Mixed**	22 (28.6)	12 (46.2)	
**Diffuse**	13 (16.9)	7 (26.9)	
**Extent of surgery**			1.000
**R0**	72 (93.5)	24 (92.3)	
**R1**	5 (6.5)	2 (7.7)	
**Nerve invasion**			0.227
**Yes**	46 (59.7)	12 (46.2)	
**No**	31 (40.3)	14 (53.8)	
**Vessel invasion**			0.023
**Yes**	52 (67.5)	11 (42.3)	
**No**	25 (32.5)	15 (57.7)	
**pT stage**			**0.015**
**T0**	4 (5.2)	0 (0.0)	
**T1**	5 (6.5)	0 (0.0)	
**T2**	9 (11.7)	1 (3.8)	
T3	43 (55.8)	12 (46.2)	
**T4**	16 (20.8)	13 (50.0)	
**pN stage**			0.483
**N0**	37 (48.0)	9 (34.6)	
**N1**	12 (15.6)	4 (15.4)	
**N2**	17 (22.1)	6 (23.1)	
**N3**	11 (14.3)	7 (26.9)	
**ypTNM**			0.143
**0**	4 (5.2)	0 (0.0)	
**I**	9 (11.7)	1 (3.8)	
**II**	34 (44.2)	10 (38.5)	
**III**	30 (38.9)	15 (57.7)	
**Harvested lymph nodes**			0.120
**Median (range)**	26 (12–80)	32 (14–60)	
**TRG**			0.317
**Grade 0**	4 (5.2)	0 (0.0)	
**Grade 1**	7 (9.1)	2 (7.7)	
**Grade 2**	27 (35.1)	7 (26.9)	
**Grade 3**	39 (50.6)	17 (65.4)	

*TRG* Tumor regression grade, *SOX* Oxaliplatin plus S-1, *PTXS* Paclitaxel plus S-1, *CI* Confidence interval

The pCR rate was 5.2% in the SOX group and 0% in the PTXS group, but there was no significant difference between the two groups (*p* > 0.05). No significant difference was found in the overall pathological response rate (TRG grade 0 + 1 + 2) between the SOX group (49.4%) and the PTXS group (34.6%) (Table 3, *p* > 0.05).

### Postoperative complications

No significant difference was observed in the overall postoperative complication rate between the SOX group (13.0%) and the PTXS group (7.7%). There was no anastomotic leakage in the SOX group and one in the PTXS group. There were no perioperative deaths in either group. The specific types of postoperative complications in the two groups are shown in Table 4.

**Table 4:** Postoperative complications between two groups

**Complication**	**PTXS (n, %)**	**SOX (n, %)**	***P*** **value**
**Overall complications**	2 (7.7)	10 (13.0)	0.280
**Intra-abdominal hemorrhage**	0	1	
**Pulmonary infection**	0	2	
**Abdominal infection**	1	1	
**Anastomotic leakage**	0	1	
**Lymphatic leakage**	0	2	
**Wound infection**	1	1	
**Catheter-related infection**	0	1	
**Remnant gastric motility disorder**	0	1	

*SOX* Oxaliplatin plus S-1, *PTXS* Paclitaxel plus S-1

### Survival analysis

**Fig. 2:**
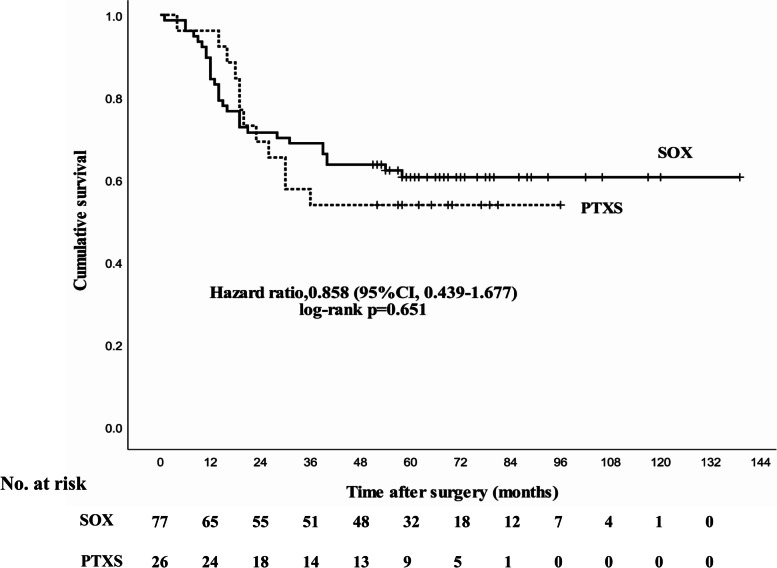
Treatment outcome of overall survival between SOX and PTXS regimens

During follow-up, there were nine patients with recurrence in the PTXS group, and 19 in the SOX group with no significant difference (*p* > 0.05). The median OS was 62 months (95% CI, 47- 76) in the PTXS group versus 92 months (95% CI, 79–105) in the SOX group. The 5-year OS rate was 55.6% in the PTXS group and 61.4% in the SOX group, with no significant difference (*p* = 0.651, Fig. 2).

**Fig. 3:**
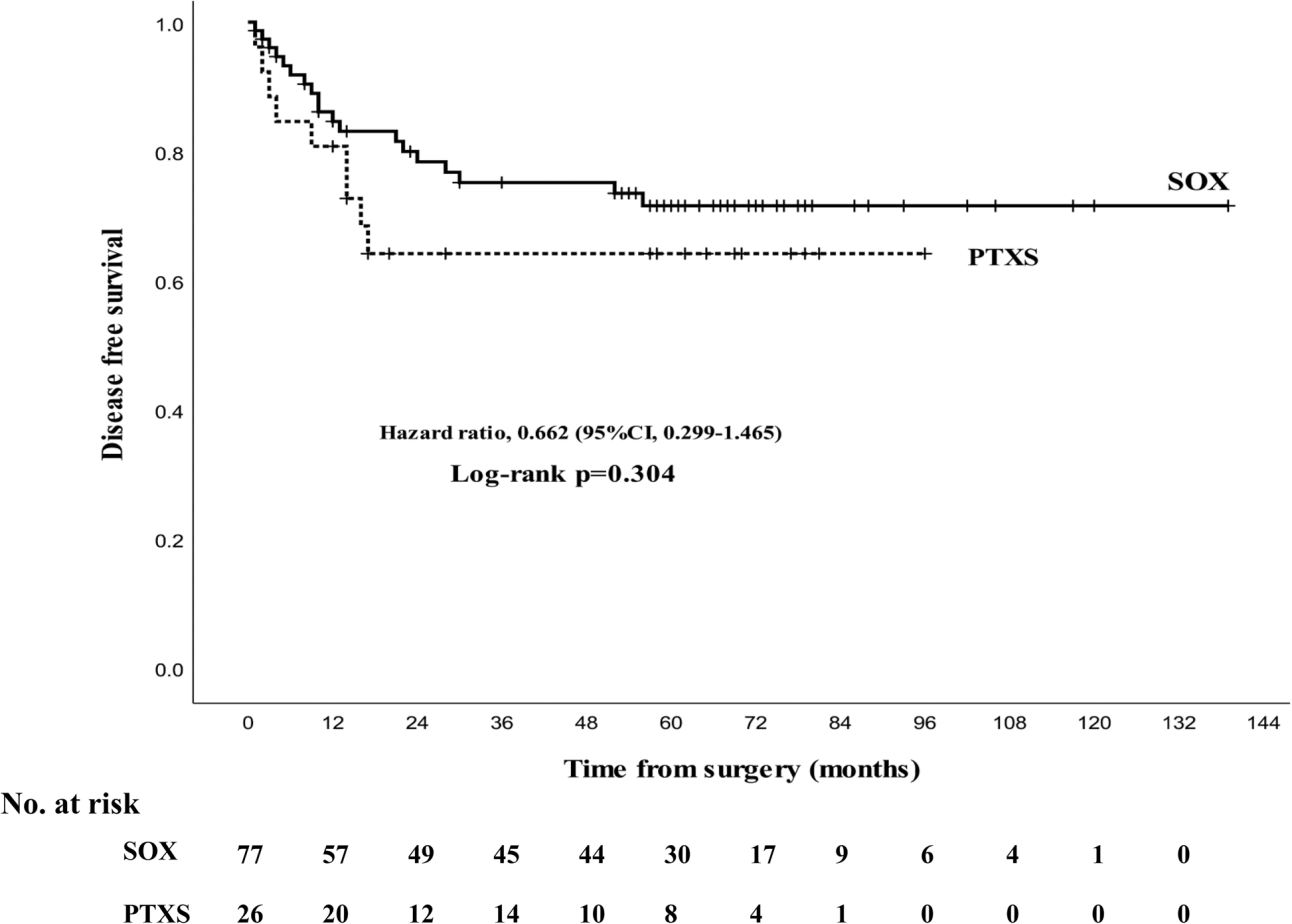
Treatment outcome of disease-free survival between SOX and PTXS regimens

There was also no difference in median DFS between the PTXS group (65 months, 95% CI, 48–81) and the SOX group (105 months, 95% CI, 91–117). The 5-year DFS rate was 66.7% in the PTXS group and 63.6% in the SOX group, with no significant difference (*p* = 0.304, Fig. 3).

## Discussion

The application value of neoadjuvant chemotherapy for locally advanced gastric cancer has reached a consensus. However, the standard neoadjuvant chemotherapy regimen is still not unified worldwide. In China, although the SOX regimen has become the first-line neoadjuvant chemotherapy regimen, some patients cannot tolerate it due to serious side effects such as neurotoxicity or myelosuppression. Chemotherapy regimens containing paclitaxel showed satisfactory efficacy and safety in the treatment of advanced gastric cancer [19, 20]. Therefore, paclitaxel has been also used in the perioperative chemotherapy regimen for LAGC [13, 21, 22]. If the PTXS regimen is not inferior to the SOX regimen in the context of neoadjuvant therapy, then the regimen can be used as an alternative to the SOX regimen.

The present study demonstrated that the proportion of downstaging rate was higher in the PTXS group (38.5%) than that in the SOX group (29.9%), although there was no statistically significant difference. Consistent with previous reports [1, 3, 4], neoadjuvant chemotherapy could achieve downstaging. Therefore, for patients with severely locally advanced gastric cancer, especially those who cannot tolerate the SOX regimen, the neoadjuvant PTXS is a desirable option.

The determination of clinical staging by CT evaluation was not very accurate. In this study, stage 0 and stage I were absent in TNM staging after neoadjuvant chemotherapy (Table 2). However, there were four patients with pathological stage 0 and 9 patients with pathological stage I in the SOX group, and one patient with pathological stage I in the PTXS group (Table 3). It was reported that the accuracy of CT in diagnosing T staging was about 77–89%, and the accuracy of diagnosing N staging was about 59- 78% [23, 24]. A prospective study (JCOG1302A) reported that there were 141 (15.2%) and 71 (7.7%) patients with pathological T1 and T2 tumors respectively among 928 patients with cT3/T4 [25]. The overdiagnosis was mainly due to the intratumoral edema or fibrosis that made the lesion look thicker on CT or endoscopic ultrasonography [25, 26]. Therefore, the efficacy of neoadjuvant chemotherapy for gastric cancer should not be evaluated solely by CT, but also combined with other tools, such as MRI or PET/CT when necessary.

This study did not reveal a statistically significant difference between neoadjuvant SOX and PTXS regimens in terms of DCR and TRG. These results indicated that the effect of PTXS as a neoadjuvant chemotherapy regimen in the treatment of LAGC was not inferior to that of the SOX regimen. Due to the synergistic cytotoxic effect of paclitaxel and fluorouracil, the significant efficacy of this regimen in advanced gastric cancer was confirmed in many clinical studies [20, 27].

The effect of different neoadjuvant chemotherapy regimens on surgical complications is also a matter of concern. This study showed that the postoperative complication rate was low and not significantly different between the two groups. Many studies showed that neoadjuvant chemotherapy did not increase the complications and postoperative mortality [1]. However, some other studies found that neoadjuvant chemotherapy increased the complication rate after gastrectomy with D2 lymphadenectomy, and reported the complication rate of patients receiving neoadjuvant chemotherapy regimen containing paclitaxel or docetaxel ranged from 17–31.3% [28–30]. Actually, gastrectomy is usually performed three to four weeks after neoadjuvant chemotherapy, at which time the effect of chemotherapy on tissue healing is negligible.

The present study also found that the 5-year OS of the PTXS group was not significantly different from that of the SOX group. Paclitaxel as monotherapy or in combination with other drugs could improve survival without compromising the quality of life (QoL) for patients with advanced gastric cancers [31]. A phage III trial comparing the efficacy and safety of paclitaxel/capecitabine (PACX) and cisplatin/capecitabine (XP) in advanced gastric cancer showed that QoL was significantly improved in PACX versus XP [19]. Baoyu Yang et al. reported that paclitaxel combined with a leucovorin and 5-fluorouracil regimen as neoadjuvant chemotherapy for gastric cancer could improve the R0 resection rate (85.2%) and 5-year survival rate (56.9%), and showed good tolerability [27].

## Conclusions

There were no significant differences between the NAC PTXS group and the SOX group in terms of clinical response, pathological response, postoperative complication rate, and 5-year survival rate. Therefore, for patients with LAGC who cannot tolerate neoadjuvant SOX, the PTXS regimen would be an ideal alternative, especially for patients with renal insufficiency. This study was a single-center retrospective trial and had its limitations. The above conclusions are insufficient and need to be verified by further multi-center randomized controlled trials in the future.

## Supplementary Information

**Additional file 1.**

## Data Availability

All data generated or analysed during this study are included in the Supplementary file 1.

## Abbreviations

PTXS: Paclitaxel plus S-1
LAGC: Locally advanced gastric cancer
SOX: Oxaliplatin plus S-1
NAC: Neoadjuvant chemotherapy
MAGIC: Medical Research Council Adjuvant Gastric Infusional Chemotherapy
EUS: Endoscopic ultrasonography
MRI: Magnetic Resonance Imaging
RECIST: Response evaluation criteria in solid tumors
CR: Complete response
PR: Partial response
PD: Progressive disease
SD: Stable disease
DCR: Disease control rate
TRG: Tumor regression grade
OS: Overall survival
DFS: Disease-free survival
DCR: Disease control rate
